# A water quality dataset of levels of metal, nutrient and anions in sample water points from sixteen selected urban and rural districts of Uganda

**DOI:** 10.1016/j.dib.2023.109601

**Published:** 2023-09-23

**Authors:** Hasifah Kasujja Namatovu, Mark Abraham Magumba, Tonny Justus Oyana

**Affiliations:** a Department of Information Systems, College of Computing and Information Sciences, Makerere University, Kampala, Uganda; b Geospatial and Computational Science Lab, College of Computing and Information Sciences, Makerere University, Kampala, Uganda

**Keywords:** Water monitoring, Aquatic informatics, Environmental science, Environmental informatics, Water *science and technology*

## Abstract

This dataset highlights some of the water quality issues in Uganda. The rationale for collecting the water samples was to test and ascertain the level and source of contamination. A total of one hundred and eighty five samples were collected from sixteen districts. At each water point, a sample was collected using a sterile plastic container, which was pre-rinsed with the water to be sampled. Water samples were drawn from protected and unprotected springs, shallow wells, taps, rain tanks, water reservoirs, open and hand dug wells and boreholes and immediately transported on ice to the National Water Quality Reference Laboratory for analysis. At the laboratory, a BWB flame photometer, Ethylenediamine tetraacetic acid (EDTA)^1^ titration and gallery plus-thermos fisher discreet analyzer were used to analyze metal, nutrient and anion elements. On-site testing of dissolved oxygen, pH, electrical conductivity and turbidity was done using a water data sonde. This data can be used to draw comparative analyses of water quality issues in rural and urban districts and help in identifying the factors that influence water quality variations. The data can further be used for trend analysis and identifying long-term patterns whilst providing insights into pollution sources and the impact of environmental and climate change. Consequently, mathematical and machine learning models can use this data together with other parameters to predict the changes in water quality which information is essential for policy and decisions making. This data can be used by environmental scientists to draw insights into the health of the aquatic biodiversity; geospatial analysts to ascertain proximal water contaminants; public health specialists to analyze pathogens leading to water-borne diseases; water chemists to study the source and cause of water pollution; data scientists to perform predictive and descriptive analyses; and policy makers to formulate laws and regulations.

Specifications Table

**Table:** 

Subject	Data Science, Environmental Science, Health and Medical Sciences
Specific subject area	Datamining and Statistical Analysis, Machine Learning, Water Science and Technology, Public Health, Environmental Informatics, Water Informatics.
Type of data	Table
How the data were acquired	The data collected involved onsite measurement of dissolved oxygen, pH, electrical conductivity and turbidity that were measured using a water data sonde calibrated on the field day using a reference temperature set at 25 °C. Sodium (Na) and Potassium (K) were measured using a BWB flame photometer while water hardness, calcium and Magnesium were determined using the standard EDTA titration method (standard methods for examination of water and waste water (APHA^a^ 2017). Anions (nitrates (NO_3_^−1^), nitrate (NO_2_-), fluoride (F-), sulphates (SO_4_^−2^), chlorides (Cl) and phosphates (PO_4_^3−^)) and ammonium in water were determined using a, gallery plus-thermos fisher discreet analyzer. A discreet analyser is an automated system for spectrophotometric and turbidimetric determination of various elements in water (ISO 15923-1). Specific amount of liquid sample is automatically dispensed into a small cuvette. Between one and three method-specific reagents are then automatically dispensed into the same cuvette. After a timed incubation, the reagents will cause color to develop in the sample proportional to the analyze concentration. The color intensity of the sample is then measured at a specific wavelength by the instrument and converted to a concentration with the aid of a blank and a calibration standard. Quality control is achieved by means of an independent standard measured in the same manner.
Data format	Raw
Description of data collection	Samples were collected in one-liter plastic bottles, labelled with identification codes, transferred into a cool box with ice packs to maintain temperatures below 10°C and transported to the National Water Quality Reference Laboratory (NWQRL)^b^. The analyzed parameters at the NWQRL included: metal elements, nutrients and anion elements relevant to water quality. A GPS location for each water point was also captured. The team drew 185 samples from water points that were more than 50 m apart because it is assumed that the spatial variations tends to differ as one moves further away from a point.
Data source location	· **Institution:** Makerere University · **City/Town/Region:** Kampala, Central Region · **Country:** Uganda
Data accessibility	Repository name: Mendeley Data. Data identification number: 10.17632/mbx7bxm4vx.4 Direct URL to data: https://data.mendeley.com/datasets/mbx7bxm4vx/4[1]

a American Public Health Association - APHA.

b National Water Quality Reference Laboratory (NWQRL).

## 1 Value of the Data

• This data is useful in understanding the degree of pollution of the different water sources. With this data, scientists and researchers are able to know the possible sources of contamination and advise accordingly.
• This dataset can be used by researchers and scientists to investigate the relationship of water quality variations in relation to environmental factors whilst evaluating the impact of water quality on public health.
• Water datasets provide a better understanding of the health of the aquatic ecosystem [2]. By analyzing certain parameters like temperature, turbidity, dissolved oxygen, pH levels and nutrient concentrations, scientists can identify the possibility of aquatic life degradation.
• This dataset is important in tracking water changes over time whilst assessing land use activities affecting water quality.
• Spatio-temporal analysis can be employed to investigate space-time variation by identifying water quality patterns persisting over time as well as running climate and water quality models to help predict weather patterns and pollution patterns.

This dataset is a source of policy and decision making by government and other policy makers. Regulations, strategies and policies can be formulated to establish quality standards and water asset development.

## 2 Objective

The data is meant to provide an insight into the current state of water quality in Uganda's water sources. From this, we envisage researchers, government agencies and other policy making organs to enforce water quality interventions to regularly monitor water. Another key objective is that, the data can be used to run mathematical and statistical models, climatic models, geospatial models and water quality models to help predict possible pollution over time. Lastly, this data was generated to help understand the extent to which land use activities affect water quality.

## 3 Data Description

This dataset describes the state of water quality in 185 water points across sixteen districts in Uganda (water quality dataset.xlsx). Details of each water point were profiled using the source name, village, parish, sub county, district, GPS location, lab identifier code and parameters tested explained in Table 1. The parameters tested included; electrical conductivity which measures the ability of water to conduct an electrical current. It is measured in microsiemens per cm (µs/cm) [3]. pH is the standard measure of how acidic or alkaline a solution is, measured in pH units [4], the allowable pH in potable water ranges between 5.5–9.5 units. Total alkalinity, measured in milligrams per liter (mg/l), is the measure of the alkalinity of substances present in water [5] that should be within the range of 100–200 mg/l. Color (Apparent), measured in platinum-cobalt (PtCo.), is the color of the whole water sample, and consists of color due to both dissolved and suspended components [2]. The permissible limits of color as per the East Africa standards 12:2018 is 50 PtCo. Total hardness is the content of salts of the alkaline earth metals calcium, magnesium, strontium, and barium measured in mg/l [6] with permissible limits not exceeding 600 mg/l. Calcium hardness, measured in mg/l is the amount of calcium ions present in the water [7] with consumable limits not exceeding 600 mg/l. Fluoride is a negative ion of the element fluorine naturally found in water, soils or food. It is measured in mg/l with permissible limits of not more than 1.5 mg/l [8]. Turbidity, measured in nephelometric turbidity unit (NTU), is the relative clarity of a liquid [9] with allowable limits for consumption not exceeding 25 NTU. Nitrates and nitrites are naturally occurring ions that are part of the nitrogen cycle with the former being the stable form of combined nitrogen for oxygenated systems and the later containing nitrogen in a relatively unstable oxidation state [10]. Both nitrate and nitrite are measured in mg/l with a maximum permissible limit not exceeding 10 mg/l and 0.9 mg/l respectively.

**Table 1:** Description of the data.

Source Name	This is the source of water. The sources in this dataset include tap / piped water from National Water and Sewerage Corporation (NWSC), a body in charge of distributing piped water in Uganda; borehole (BH); shallow wells (SW); protected spring and open well; unprotected springs, water harvested from rain (rain tank) and hand dug well.
Village	This is the lowest administrative unit with approximately 70 households.
Parish	A Parish is the next administrative unit from a village made up of villages.
Sub-County	A sub-county is made up of several parishes
County	A county is made up of several sub-counties
District	A district is made up of several counties
GPS Location	This is the spatial location of each water point
Lab identifier code	Each water sample was given a unique identifier to differentiate them
Parameter	These are the metals, nutrients and anion elements that were being tested
Units	Standard used in measurements
Potable water standards (DEAS12:2018 Maximum permissible for Natural potable Water)	Potable water is all water either in original state or after treatment that is intended for human drinking, cooking, food preparation, domestic purposes, food production regardless of its origin [13] Potable water standards (DEAS^a^ 12:2018) sets minimum requirements for physical, chemical and micro-biological characteristics affecting safety and quality of drinking water [13].

a Draft East African Standards - DEAS.

Sulphates are a combination of sulfur and oxygen which are part of naturally occurring minerals in some soil and rock formations that contain ground water [11]. Sulphates are measured in mg/l with permissible limits not exceeding 400 mg/l. Ammonium is a diffusion of ammonia gas with acceptable maximum limit in potable water of 0.5 mg/l as per the East African Standard 12:2018. Chlorides are major anions found in all natural and waste water [12]. They are measured in mg/l with consumable limits of not more than 250 mg/l. Phosphates are chemical compounds containing phosphorus, a non-metallic element found in rock as inorganic phosphates equally found in fertilizers and detergents [3]. Sodium and potassium, measured in mg/l, are alkaline metals often found in ground water [4,5] with permissible limits of consumption not exceeding 200 mg/l and 50 mg/l respectively. Iron and manganese are metallic elements present in many rocks, soils and minerals [6] while total iron is free iron comprising iron hydroxide and iron complexes [7]. Measured in mg/l, these metals have a maximum allowable limit for consumption of 0.5 mg/l, 0.1 mg/l and 0.5 mg/l respectively. Free chlorine is the amount of chlorine that has yet to combine with chlorinated water to sanitize contaminants whereas total chlorine is a sum of combined chlorine and free chlorine [8]. These two components are measured in mg/l. Escherichia coli (E.coli) is a fecal coliform bacteria found in the environment, intestines of animals and people which can get into contamination with water [9]. E. coli is measured in colony-forming unit (CFU/100 ml) with allowable consumption limit of not more than 1CFU/100 ml. Total dissolved solids (TDS) are organic matters and inorganic salts including potassium, sodium, calcium, magnesium, bicarbonates, chloride, and sulphates that are dissolved in water [10]. TDS is measured in mg/l, with permissible limits not exceeding 1500 mg/l.

Bicarbonates are formed by a dissolution of carbonate (magnesium and calcium) and alumino-silicate minerals by carbondioxide and water [11]. They are measured in mg/l with the highest permissible limits not exceeding 400 mg/l. Calcium and magnesium occurs naturally in water dissolving from rock such as marble, limestone, calcite, gypsum, dolomite, fluorite and apatite [12]. They are measured in mg/l with consumable limits not exceeding 150 mg/l and 100 mg/l respectively. Magnesium hardness, measured in mg/l, is the presence of magnesium ions in water [13], whose consumable limits as per the East African Standards shouldn't exceed 600 mg/l. Silica, which is also measured in mg/l comes from the weathering of silicate minerals in the ground that mixes with water [12]. The permissible limits should not exceed 30 mg/l. Mercury is a silver-white metal found in small amounts in the earth's crust that can get in water through several ways like rain and snow [14]. This metal in measured in mg/l with allowable limits for consumption not exceeding 0.001 mg/l. Lead is a colorless metal that gets in water through contact with lead containing materials such as household plumbing [15]. It is measured in mg/l whose permissible limit in water should be less than 0.01 mg/l. Dissolved oxygen is the measure of how much oxygen is dissolved in the water i.e. the amount of oxygen available to living aquatic organisms [16]. It is measured in mg/l. Field temperature was the temperature that was recorded at the time when the water samples were being drawn.

Each excel sheet in the dataset presents results of each district. The districts include Jinja, Bugiri, Iganga, Mukono, Luwero, Nakaseke, Nakasongola, Buikwe, Kamuli, Kayunga, Kamwenge, Lamwo, Luuka, Bugweri, Mubende and Wakiso. For each result set, the last column represents the standards (DEAS 12:2018) against which, each parameter is measured. ND (not detectable) as represented under total and free chlorine means the results were below the detectable limits. TNTC (too numerous to count) implies that the bacterial count exceeded 1000.

Details of the tested parameters i.e. metal, nutrient and anion elements can be found in the repository https://data.mendeley.com/datasets/mbx7bxm4vx/4 [1]

## 4 Experimental Design, Materials and Methods

Water samples were collected from sixteen administrative units (districts) between January and March 2023. A total of one hundred and eighty five water points were considered for the survey across the sixteen districts as demonstrated in Table 2 below. The choice of the central region was based on the fact that, it is the most populous, urban and an industrial region in Uganda, hosting the capital city, Kampala. The east and the north are the most agricultural [14] and the west is predominantly a cattle corridor. The choice of the districts within a region was based on, remoteness of the district (rural or urban), economic activity, population and water source. We used a snowballing technique to identify water points. Water samples were drawn from boreholes, protected and unprotected springs, taps, hand dug wells, shower and open wells and rain tanks for physio-chemical testing. Sample points were selected based on the proximity to a possible pollution source, degree of human activity in the surrounding areas, population served and whether the facility was functional. Some areas had dilapidated boreholes and non-functional water sources which explains the variation in the number of water points that were considered in this study. At the point of water abstraction, once a water source was found to be non-functional, common to boreholes, then a next source was considered.

**Table 2:** Samples collected in each district.

Districts	Number of water points	Number of parameters tested	District Status
**Central**			
Mukono	8	17	Urban
Luwero	14	20	Rural
Nakaseke	17	20	Rural
Nakasongola	9	20	Rural
Kayunga	10	22	Urban
Wakiso	9	25	Urban
Buikwe	8	22	Rural
Mubende	2	27	Rural
**East**			
Iganga	9	17	
Bugiri	13	17	Rural
Jinja	11	17	Urban
Kamuli	15	22	Rural
Bugweri	2	27	Rural
Luuka	5	11	Rural
**North**			
Lamwo	34	14	Rural
**West**			
Kamwenge	19	10	Rural

## 5 Water Quality Analysis

A full profile testing was conducted on all the water samples at the National Water Quality Reference laboratory at Entebbe. The following parameters were tested in the laboratory: potassium (K), sodium (Na), total dissolved solids (TDS), total hardness as CaCO3, calcium (Ca), magnesium (Mg), bicarbonates (HCO_3_-), fluoride (F-), sulphates (SO_4_^−2^), chlorides (Cl), nitrates (NO_3_^−^), nitrate (NO_2_-), ammonium (NH_4_^+^), phosphates (PO_4_^3−^), manganese (MnO_2_), iron (Fe), silica (SiO_2_), mercury (Hg), lead (Pb) and fecal coliforms. Temperature, pH, dissolved oxygen (DO), electrical conductivity (EC) and turbidity were measured insitu using water data sonde (Model; Eureka Water Probes Manta+ 35) calibrated on the field day using a reference standard (Hanna HI9828–25) at temperature set at 25 °C, which is within the ambient conditions.

Total dissolved solids were derived from electrical conductivity using a factor of 0.7. Total and free chlorine were determined onsite following diethyl‑p-phenylene diamine (DPD) chlorine method and using a Lamotte DC1500 CL chlorine calorimeter from LaMotte USA. The calorimeter comes with a factory calibration and calibration validity is checked prior to use. Duplicate samples were collected in one-liter plastic bottles with the second sample being acidified with 1% nitric acid at pH of <2 for the analysis of heavy metals. The collected samples were labelled with identification codes, and transferred into a cool box with ice packs to maintain temperatures below 10 °C and transported to the NWQRL www.nwqrl.mwe.go.ug. The analyzed parameters at the NWQRL included: metal elements, nutrients and anion elements relevant to water quality. The standard methods for the examination of water and waste water (APHA 2017) and related ISO standards were used for the range of analysis of parameters for this study. All reagents used were analytical grade from reputable suppliers including thermoscientific and Sigma Aldrich while all equipment used in this study were calibrated with an acceptable regression coefficient (R^2^) set at 0.999 or better.

Physical water quality parameters were determined on the collected samples including; color was determined spectrophotometrically at 450 and 465 nm, with platinum-cobalt solution as standards using a Hach UV VIS Spectrophotometer (Model; DR.. 6000 with preprogrammed water analysis methods including color). Turbidity was determined by Nephelometric Method using a Tungsten Lamp Turbidimeter (Model; Hach TL2350) with reference standards from both the equipment manufacturer (Hach StablCal Calibration set Cat 2659505) and Sigma Aldrich.

Major anions (Fluorides (F); Chlorides (Cl); Nitrites (NO_2_-N); Nitrates (NO_3_-N), sulphates (SO_4_), phosphates (PO_4_-P)), and Silica (SiO_2_) as well as Ammonium (NH_4_-N) were performed on a discreet photometric analyser (Model; Thermoscientific gallery plus). The fully automated discreet analyser provided quality repeatable water analysis results with minimal errors, thus ensuring confidence in the quality of analytical results. For each parameter on a sample, a preset volume of a sample and reagent are mixed into a discrete cuvette, incubated for a color reaction to take place and at the end of the incubation period, the absorbance of the solution is measured at a known parameter wave length and converted to concentration by means of a calibration curve for each parameter.

Except for chlorides that used a 2nd Order curve, all other parameter calibrations were 1st order using 100 mg/L calibrants in automatic dilution function prepared from 1000 mg/L NIST traceable thermoscientific primary standards for each individual parameter. Only calibration curves with a coefficient of determination (R^2^)=0.999 or higher were used in data generation to ensure acceptable accuracy. On the discrete analyser, Chlorides were determined following the standard Mercury (II) thiocyanate method (SM 4500 Cl-E) where Chloride reacts with mercury (II) thiocyanate to form non-ionized (but soluble) mercury (II) chloride and an equivalent amount of free thiocyanate, which forms a red complex with iron (III). The absorbance at 480 nm is a measure of the chloride content.

Ammonium was determined as ammonium nitrogen using the Sodium dichloroisocyanurate, sodium salicylate and sodium nitroprusside method at an absorbance at 660 nm (ISO 7150). While nitrates and nitrites were respectively determined by the hydrazine (SM 4500-NO3- H) and Sulfanilamide coupling (SM 4500 NO2-B), method. Sulphates were analysed by the standard barium chloride method (SM4500 SO4- E), flourides by Alizarine fluorine blue and cerous nitrate method (SM 4500 F-E) and phosphates by its reaction with molybdate and antimony potassium tartrate in an acid environment and subsequent reaction by ascorbic acid (SM 4500 P-E).

Major cations (calcium and magnesium) and hardness were determined by the standard EDTA titration method using Erichrome Black T as an indicator for total hardness and Murexide as an indicator for calcium determination. A pure reagent of calcium carbonate was used for quality control. Sodium and potassium were determined by flame photometry (Model BWB flame photometer with a five-channel instrument from BWB technologies UK). The instrument provides for simultaneous detection and display of sodium and potassium concentration in a water sample based on an already generated calibration curve.

Alkalinity determination was based on the standard acid base titration (SM 2320-B) using Bromocresol green Indicator solution that turns from blue to gray at pH 4.5. While bicarbonates were calculated from alkalinity using an alkalinity bicarbonate calculator. Total Iron was determined following a 1,10–Phenanthroline method which is based upon spectrophotometric measurement at 510 nm of the colored complex formed by the reaction between ferrous iron and 1,10 – Phenanthroline after the reduction of iron to the ferrous state.

Metals (mercury, lead and Manganese) were simultaneously determined on an inductively coupled plasma-Optical emission spectrometer (ICP-OES, model: Perkin Elmer Optima 7000 DV).

Microbial analysis (*Ecoli and Total Coliforms*) was undertaken using mobile membrane filtration kits (Wagtech AQUASAFE WSL50 PRO) and compact dry media (06743) from Nissui Pharma, Japan. The compact dry media used is able to isolate E.Coli and Total Coliform bacteria in a water sample by way of bacteria colony growth and enumeration after incubation at 37 °C for 18 h and color development (Blue colonies for E.coli and Pink for Total Coliforms).

## Ethics Statements

This work does not contain studies involving human, animal or social media data.

## CRediT authorship contribution statement

**Hasifah Kasujja Namatovu:** Conceptualization, Methodology, Validation, Investigation, Data curation, Writing – original draft, Writing – review & editing, Supervision, Project administration, Funding acquisition. **Mark Abraham Magumba:** Data curation. **Tonny Justus Oyana:** Conceptualization, Methodology, Writing – review & editing, Supervision, Project administration.

## Data Availability

Water Quality in Selected Districts in Uganda (I-WAQ) (Original data) (Mendeley Data)
